# Structural mechanism of DNA-mediated Nanog–Sox2 cooperative interaction†

**DOI:** 10.1039/c8ra10085c

**Published:** 2019-03-13

**Authors:** Dhanusha Yesudhas, Muhammad Ayaz Anwar, Sangdun Choi

**Affiliations:** Department of Molecular Science and Technology, Ajou University Suwon 16499 Korea sangdunchoi@ajou.ac.kr +82 31-219-1615 +82 31-219-2600

## Abstract

The efficiency of stem cell transcriptional regulation always depends on the cooperative association and expression of transcription factors (TFs). Among these, Oct4, Sox2, and Nanog play major roles. Their cooperativity is facilitated *via* direct protein–protein interactions or DNA-mediated interactions, yet the mechanism is not clear. Most biochemical studies have examined Oct4/Sox2 cooperativity, whereas few studies have evaluated how Nanog competes in the connection between these TFs. In this study, using computational models and molecular dynamics simulations, we built a framework representing the DNA-mediated cooperative interaction between Nanog and Sox2 and analyzed the plausible interaction factors experienced by Nanog because of Sox2, its cooperative binding partner. Comparison of a wild-type and mutant Nanog/Sox2 model with the Nanog crystal structure revealed the regulatory structural mechanism between Nanog/Sox2–DNA-mediated cooperative bindings. Along with the transactivation domains interaction, the DNA-mediated allosteric interactions are also necessary for Nanog cooperative binding. DNA-mediated Nanog–Sox2 cooperativity influences the protein conformational changes and a stronger interaction profile was observed for Nanog-Mut (L103E) in comparison with the Nanog-WT complex.

## Abstract 2

The efficiency of stem cell transcriptional regulation always depends on the cooperative association and expression of transcription factors (TFs).

## Graphical abstract

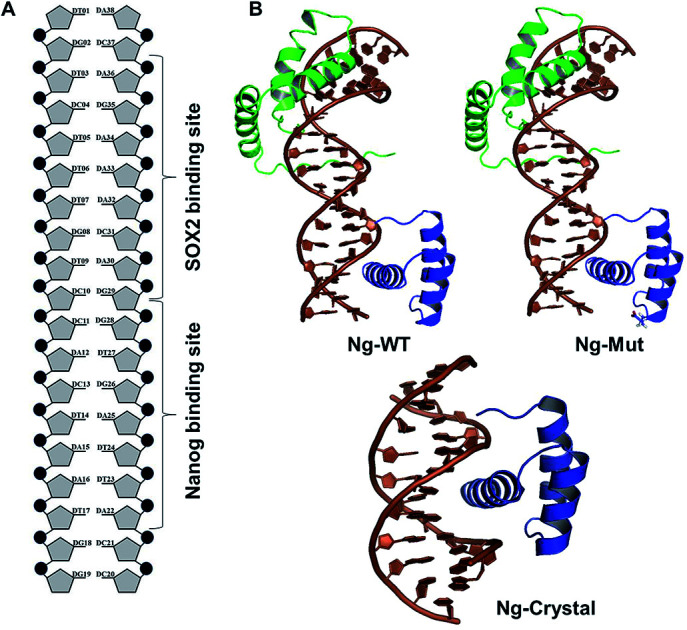

## 1 Introduction

Nanog is a homeodomain protein that appears to function at the top of a regulatory circuitry necessary for development processes and stem cell pluripotency.^1^ The 305 amino acids of the Nanog polypeptide have three functional domains: a serine-rich N-terminal domain (ND), central homeodomain (HD), and C-terminal domain (CD).^2^ The homeodomain consists of 3 helices, of which helices 2 and 3 (H2 and H3) form a helix–turn–helix motif. Helix 3, also known as the recognition helix, is inserted in the major groove of DNA and is primarily responsible for interacting with the bases; additional base contacts are formed by the N-terminus of the homeodomain, which reaches into the minor groove.^2^

The self-renewal efficiency of embryonic stem cell (ESC) is determined by the Nanog protein level expression. However, how Nanog is regulated at the protein level and the protein partners of Nanog that function to direct self-renewal are largely unclear.^3,4^ Based on experimental studies, more than 130 proteins (including transcription factors [TF], chromatin modifying complexes, phosphorylation and ubiquitination enzymes, basal transcriptional machinery members, and RNA processing factors)^5,6^ made a Nanog interactome, and Sox2 was identified as a healthy interacting partner of Nanog. Nanog-bound promoters are co-occupied by the octamer binding protein 4 (Oct4) and SRY-related HMG-box gene 2 (Sox2) proteins.^2^ Nanog and Sox2 are mainly interacting *via* their transactivation domain (TAD), whereas modeling and simulating the unstructured TAD domain residues (∼250) of both the proteins is a tedious process.

To date, biochemical characterization of protein–protein interactions in pluripotent cells has been studied extensively for Sox2 and Oct4.^6–8^ From a biochemical as well as computational perspective, little is known about how Nanog fits into the tight relationship between Oct4 and Sox2.^9^ Hutchins *et al.* (2013) described a *de novo* motif representation for the Nanog–Sox2 complex. They developed a tool for systematically evaluating ChIP-seq data (from mouse ESCs) to identify TF composite motifs and found that the Nanog–Sox2 motifs are in proximity to each other.^10^ ChIP-seq peaks of the Nanog–Sox2 motif have been observed in *Zfp42*, *Klf5*, *Ncam1*, and *Myst4*.^6^ Since there is no crystal structure showing the direct physical interaction of Nanog–Sox2, we modeled the complex based on the motif representation^10^ described by Hutchins *et al.* The model system actively included the mutant L122E, which enhances protein stability and DNA-binding affinity.^10,11^ Comparative study of the wild-type and mutant Nanog–Sox2 model systems against the Nanog crystal structure reveals the cooperative protein–protein and protein–DNA mediated interactions.

Molecular dynamics (MD) simulation is suitable for exploring the mechanism of a protein–protein/protein–DNA interface.^12^ Therefore, we conducted comparative studies of the hypothesis models (Nanog–Sox2 partnership) and Nanog crystal structure using MD simulation. Our results revealed the structural mechanism and changes of Nanog that are influenced by its cooperative binding partner, Sox2.

## 2 Methods

### 2.1 Molecular system

**Fig. 1:**
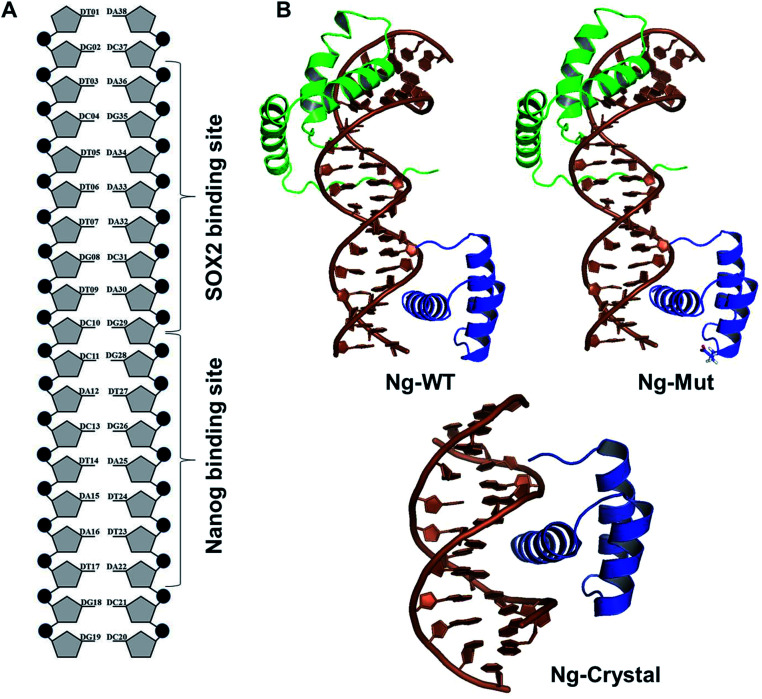
Structural organization of the complexes. (A) DNA organization labeled with corresponding Sox2 and Nanog binding sites. (B) The structural models of Ng-WT, Ng-Mut (mutated residue has been shown in stick representation), and Ng-Crystal have been presented. DNA bases are in brown, filled-circle shape, Sox2 has been represented as green, and Nanog is in blue. All the structures are with reference to Nanog binding with DNA.

To study the DNA–Nanog/Sox2 interactions, three systems were defined for this study: Ng-WT (wild-type), Ng-Mut (L122E mutated), and Ng-Crystal (crystal structure). The starting structures of Nanog and Sox2 for these models were obtained from protein data bank (PDB) IDs 4RBO^11^ and 1GT0 ^13^ respectively. The DNA sequences have been extracted from the CHIPSeq data for Sox2 and Nanog binding motif as reported by Hutchins *et al.*, 2013.^10^ Based on this reference Sox2_0 bp_Nanog (the highest *Z* score value) CHIPSeq data, we have modeled our target complexes. The binding site of Sox2 (C(T/A)TTGTT) and the binding site of Nanog (TAAT(G/T)(G/T)) are having the variable binding bps in their binding site. Once the binding motif has been confirmed, the ternary complex has been built by taking 1GT0 as a reference that share motif similarity, as well as represent the organization of ternary complex of Sox2 with Oct4, and the Oct4 was replaced with Nanog. Since Oct4 has HMG and HD domains in its crystal structure, it is easy to superimpose the Nanog HD domain in the place of Oct4 HD domain and the corresponding DNA bases were replaced by Nanog binding site. Thereby the final modeled complex for Sox2–Nanog has 
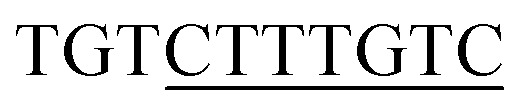
^14^ for Sox2 and 
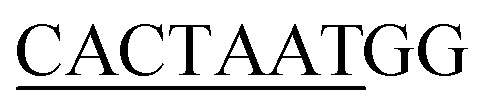
 for Nanog (underlined region represents the respective binding site) with 0 bp gap (TGTCTTTGTCCACTAATGG) between them. The two bp on each side of dsDNA are random and have been appended to mimic the full-length DNA (Fig. 1). Thus, the final DNA fragment was 20 nucleotides long, containing the Nanog (numbered from 1–80) and Sox2 (numbered from 81–130) protein molecules. Protein modeling and DNA bp alteration were conducted using the Discovery studio visualization package. All protein residues were in their default protonation states at neutral pH. The systems were solvated in an orthorhombic box of 22 668 water molecules. Sodium and chloride ions were added to neutralize the systems up to a final concentration of 150 mM.

### 2.2 MD simulation

The systems were equilibrated by 1100 steps of energy minimization, followed by a 100 ps MD simulation in the NVT ensemble and for 100 ps in NPT ensemble. The simulation was conducted in GROMACS v5.0.7 ^15^ with AMBER-99SB-ILDN force field^16^ and a 200 ns production run for each system (total 3 × 600 ns) was carried out using the NPT ensemble. The TIP3 model was used for water molecules. The temperature was maintained at 300 K by Langevin dynamics.^17^ Periodic boundary conditions were applied, and the pressure was kept at 1 atm by the Nosé–Hover Langevin method. A 9 Å cut-off was used for the van der Waals interactions. Electrostatic forces were computed by the Particle Mesh Ewald algorithm^18^ with a maximum grid spacing of 1.0 Å. Bonds with hydrogen atoms were restrained by the LINCS algorithm using a time step of 2 fs.^19^ The detailed protocol has been described previously.^20,21^

### 2.3 Principal component analysis

Principal component analysis (PCA) provides a complete picture of conformational flexibility by calculating the correlated motion of atoms in a protein–DNA complex. This technique is based on constructing a covariance matrix of complex sets of variables.^22–25^

The formula for covariance matrix with elements *C*_*ij*_ for coordinates *i* and *j* is given as

1 *C*_*ij*_ = 〈(*X*_*i*_ − 〈*X*_*i*_〉)(*X*_*j*_ − 〈*X*_*j*_〉)〉

where *X*_*i*_ and *X*_*j*_ are the mass-weighted coordinates of the atoms present in the system and 〈〉 is the average of all structures sampled during the simulations. The eigenvectors represent the direction of coordinated motion of atoms and the eigenvalues represent the magnitude of the motion along the direction.^23^

### 2.4 Quasi-harmonic entropy calculation

Conformational entropy from MD simulation trajectories was performed by quasi-harmonic analysis. Along with Schlitter's heuristic formula, diagonalizing the covariance matrix to obtain quasi-harmonic frequencies from the eigenvalues provides a simplified account of the dynamic behavior of a molecule in a subspace.^26^ Schlitter's method estimates the absolute configurational entropy of a macromolecule from a covariance matrix of the Cartesian coordinates of atoms calculated by molecular dynamics simulations.^27^ The Schlitter equation combined with quasi-harmonic analysis was used to estimate the changes in conformational entropy in the protein–DNA complex, contributing to understand the thermodynamic properties of a system. Entropy was estimated from covariance matrices of Cα atom fluctuations observed during the simulations based on the quasi-harmonic approximation.

### 2.5 DNA parameter analysis (CURVES+)

CURVES+ ^28^ tool was used to analyze DNA parameters and is a simple matrix-based scheme for calculating a complete set of parameters. Equally spaced 400 snapshots of DNA extracted from the whole trajectory were considered as inputs. Average values on intra- and inter-base pair nucleotides were calculated for the DNA parameters.

## 3 Results

### 3.1 Structural analysis

To evaluate the structural mechanism of the DNA-mediated Nanog–Sox2 cooperative interaction, the 3 complexes were created that have been referred to as follows: Ng-WT is the wild-type Nanog bound to DNA along with Sox2, Ng-Mut is identical to Ng-WT except for one residue, L103E mutation in Nanog (crystal structure numbering is L122), and Ng-Crystal is the native Nanog crystal structure without Sox2 (Fig. 1). To examine the cooperativity of binding partners at the atomic level, it would be worthwhile to compare the modeled systems (Ng-WT and Ng-Mut) with the native Nanog crystal structure (Ng-Crystal) (Fig. 1). All three systems were independently simulated thrice for a span of 200 ns each, and the average results are illustrated below.

**Fig. 2:**
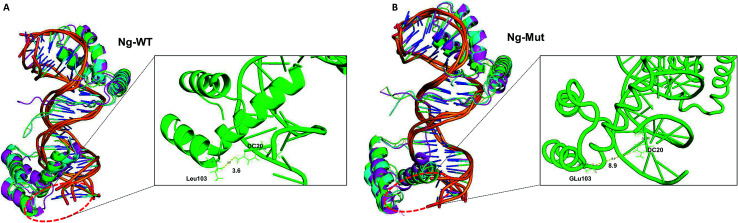
Structural comparison at varying time intervals. Superimpose structure of Ng-WT (A) and Ng-Mut (B) complexes in initial (green), intermediate (cyan) and final state (magenta) conformations are represented. The minimum distance between the mutant residue (L103E) and its nearby DNA base pair is also marked.

The root mean square deviation (RMSD) of the backbone atoms of the Ng-Crystal was constant throughout the simulation, whereas the Ng-WT and Ng-Mut complexes showed deviation in their backbone atoms (Fig. S1†). The superimposed structures of first, last and intermediate snapshots were displaying the fact that in both complexes, Nanog structures were moving towards DNA for better interaction (Fig. 2). The minimum distance between Nanog and Sox2 fluctuated from around 3.3 to 3.5 nm for both the complexes (Fig. S2†). Even though the distance between Sox2 and Nanog was ∼3.4 nm, the hydrogen bond interactions between Nanog and Sox2 were observed to be zero in Ng-WT, whereas in Ng-Mut only one interaction had been observed between Arg76 of Sox2 with Gln135 of Nanog. However the mutant residue E103 does not take part in any of the interaction with Sox2 (Table 1). Table 1 lists the residues that are making both protein–protein and protein–DNA interactions in all three systems. The subtle difference between the number of hydrogen bonds are critical as the energy contribution for individual hydrogen bond can be from −1.5 kcal mol^−1^ per hydrogen bond that can be translated to a roughly 10-fold difference in probability of two conformations.^29,30^ Thus, the breakage/formation of a single hydrogen bond has profound effect on protein–DNA stability.

**Table 1:** Interacting residues in protein–protein and protein–DNA interactions^a^

**Ng-WT (Sox2–Nanog)**					Nil				
**Ng-Mut (Sox2–Nanog)**					*ARG76*	*NH1*	*GLN135*	*O*	*3.232* Å
**Ng-WT (Sox2–DNA)**					**Ng-WT (Nanog–DNA)**				
SER31	HB1	DC4	O2	2.542	SER31	HB2	DT5	O4′	2.706
LYS35	HZ1	DT5	H5′	3.142	SER34	HB2	DT5	H2′	2.403
SER31	HB2	DC4	H1′	3.34	LYS35	HG1	DT5	H2′	3.468
LYS35	HE2	DT6	O1P	2.316	GLY38	CA	DT6	H4′	3.215
PHE10	HZ	DT6	H	2.889	MET11	HE3	DT6	O2	3.232
MET11	HE2	DT6	H3	3.417	PHE10	CZ	DT6	H2′	3.035
GLY38	HA2	DT6	H2′	3.334	GLY38	HA2	DT7	H5′	3.418
LYS42	HZ3	DT7	O1P	3.28	TRP41	HD1	DT7	H5′	2.936
MET11	HE1	DT7	O2	2.806	TRP41	HD1	DT7	C4′	3.053
TRP41	HD1	DT7	C3′	3.327	ASN8	ND2	DT7	O2	3.356
ASN8	HD2	DG8	C1′	3.397	ARG5	NH1	DG8	N2	3.188
MET11	HE2	DA32	N3	3.447	TYR72	HE2	DC10	O2	2.546
MET7	HG1	DA32	H2′	3.352	TYR72	HD2	DC10	O2	3.225
LYS4	HZ2	DA33	P	2.359	PRO74	HG2	DC10	O2	3.104
ASN30	H	DA36	O	2.88	ARG76	HH2	DT12	P	2.423
HIS29	HD2	DA36	C1′	3.033	LYS77	HZ3	DG29	H4′	3.35
HIS29	HD2	DA36	C4′	3.296	PRO74	HG2	DG29	N2	3.073
MET28	C	DA36	H	3.466	ARG75	O	DA30	C4′	3.241
**Ng–Mut (Sox2–DNA)**					**Ng–Mut (Nanog–DNA)**				
SER31	HB1	DC4	H1′	3.342	LYS121	HZ2	DG8	O2P	1.775
LYS35	HE2	DT5	H5′	2.273	GLN125	HE2	DG8	C2′	3.041
SER31	CA	DT5	H1′	3.007	TYR100	HE2	DT9	O1P	3.277
SER34	HB2	DT5	C1′	3.277	ARG128	HH2	DT9	O2P	1.839
LYS35	HE2	DT5	C3′	3.293	GLN125	NE2	DT9	O2P	2.746
GLY38	HA1	DT6	C4′	3.448	ARG128	HE	DT9	O2P	2.201
PHE10	CE2	DT6	H1′	3.245	GLN125	HG2	DT9	C5	3.44
SER34	HB1	DT6	H1′	2.76	MET129	SD	DC10	O5′	3.373
ARG5	HD2	DC31	H4′	2.088	MET129	HE1	DC10	H3′	2.63
TYR72	CE1	DC31	H4′	3.139	ARG133	HD2	DT24	H5′	3.246
ARG75	HH2	DC31	O2P	2.407	LYS130	CE	DC25	O2P	3.259
HIS29	ND1	DC37	H4′	3.383	ARG133	HD2	DC25	O2P	3.341
ASN30	HB1	DA36	O4′	3.256	LYS130	HZ3	DC25	O5′	3.367
ARG75	NH2	DC31	O5′	3.341	TRP123	HD1	DA26	O2P	2.875
TYR72	CE1	DC31	H5′	3.372	THR81	H1	DA26	H4′	2.54
TYR72	HE1	DC31	H5′	2.318	GLN119	NE2	DA26	H3′	3.437
ARG75	HE	DC31	P	2.518	THR81	H2	DA26	O3′	2.847
					GLN119	HE2	DA27	O1P	3.305
					THR122	HG2	DA26	C8	3.186
					ASN126	HD2	DA27	H61	3.193

a The protein residues (from Sox2 and Nanog) with the base pairs interactions have been tabulated, and the interacting distances are in Å. The protein–protein interaction marked with *italic*.

Furthermore, the lowest energy structure was taken based on the free energy landscape (FEL) energy values, and the interaction patterns were observed (Fig. S3†). The importance of the mutant residue (L103E) was monitored very carefully by including the simulation of Ng-Crystal–Mut structure also (Fig. S4 and S5†). The radius of gyration shows that the Ng-Crystal–WT complex was observing very high compact conformation compared with the Ng-Crystal–Mut complex; same has been reflected in the RMSF profile also (Fig. S4†). The interaction profile of L103E residue against DNA was observed and it was evident that for Ng-Crystal–Mut and Ng-Crystal–WT complexes alone was experiencing a direct interaction of E103 with the DNA bases at less than 4 Å, whereas the other complexes (Ng-WT and Ng-Mut) didn't observe this specific interaction (Table 1 and S1†). Although the minimum distance between Nanog and DNA was ∼2.8 nm for both the complexes (Fig. S5A†), both Ng-WT and Ng-Mut failed to make direct interaction between E103 and DNA base pair.

### 3.2 Residual movement

Both the Ng-WT and Ng-Mut complexes showed dominant movement with respect to DNA sequence and the movements were distinct for each complex. The relative movement of the protein along the DNA was identified by examining the positions of helix 3 residues with respect to the plane of the DNA bases (Fig. S6 and S7†). Because helix H3 formed an extensive DNA contact interface in the major groove, the interactions of helix H3 residues were considered critical for determining the specificity to the core consensus sequence. Sox2 binding altered the Nanog conformation along with its DNA. Therefore, even if the Nanog protein remains bound to the DNA molecule for the entire MD trajectory, it would not be fixed at a specific DNA sequence site and consequently, facilitate non-specific binding. Therefore, the protein was moving and sampled at least two or three different base pair sequences.

The alignment of charged residues from Nanog helix H3 with the plane of the DNA was observed and distinguished throughout the simulation. In the Ng-WT system, all residues (K118, T122, and Q125) surveyed the A15, A16, and T17 bp sequences except M129 residue (Fig. S6†). The M129 residue showed a stronger interaction with A16 bp, and thus its movement to other bp sequences was restricted. In contrast, in the Ng-Mut system, more residues from helix H3 (K118, Q119, K121, T122, Q125, R128, and M129) interacted with its corresponding DNA bps, and their movements towards the DNA bp sequences were higher compared to Ng-WT (Fig. S7†).

### 3.3 Mapping of protein conformational changes

**Fig. 3:**
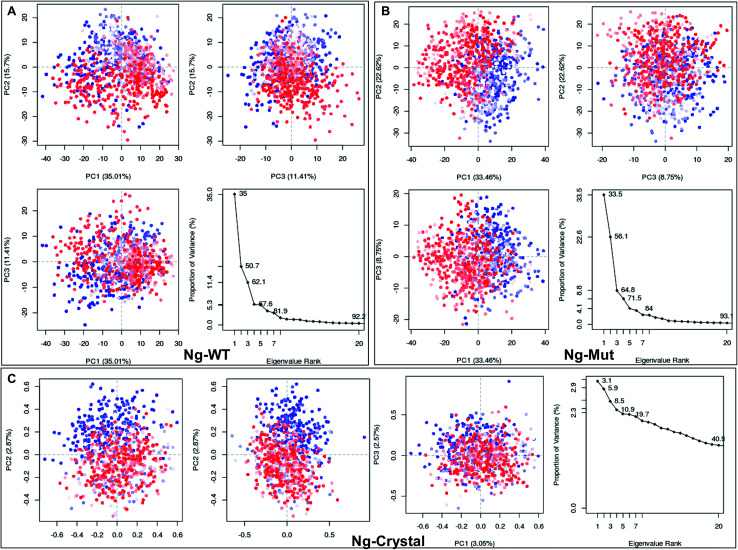
Mapping of protein conformational changes. Representation of the conformational changes of heavy atoms of Nanog in Ng-WT (A), Ng-Mut (B), and Ng-Crystal (C) using principal component analysis (PCA) by plotting their eigenvectors 1, 2 and 3. These vectors have been obtained by first removing the translational and rotational movements and then constructing the covariance matrix. Representation of the individual as well as the collective motion of eigenvectors with the cumulative percentages for Ng-WT, Ng-Mut, and Ng-Crystal are indicated. The blue and red dots denote the initial and final conformational switch of the complexes, the intermediate state is represented by white dots.

Structural flexibility of a protein has been correlated with different biological functions. To better understand the conformational changes of Nanog protein influenced by Sox2 binding, the MD trajectories of the Ng-WT and Ng-Mut systems were evaluated by principal component analysis (PCA) (Fig. 3). PCA plots show the trajectory frames onto the lowest frequency eigenvectors, and the first few eigenvectors account for most protein motions and capture large-scale motions.^31^ The first 10 eigenvectors greatly contributed to the collective motions; the collective modes of each system with their cumulative percentages of 92, 93, and 40 for Ng-WT, Ng-Mut, and Ng-Crystal, respectively, are shown in Fig. 3. Each trajectory position was plotted as the dot product of the coordinates and eigenvector, representing the range of displacement along each eigenvector from the average position. The distribution of eigenvector values corresponding to the protein motions in the essential subspace has been provided with that clusters of representatives explored tertiary conformations. The red and blue color represents the final and initial conformational clusters during the simulations. The white color dot represents the intermediate state. The projection of the trajectories on the plane defined by the first, second and third eigenvectors indicated that Ng-WT, and Ng-Mut exhibited no energy barrier between their metastable states which indicated that no energy penalty required to switch from one conformation to another. In general, Ng-WT and Ng-Mut have more of wider conformational basins than the single wider basin of crystal structure (Fig. 3C).

**Fig. 4:**
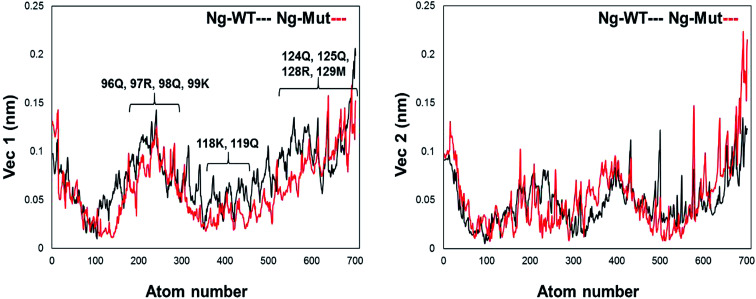
Residue fluctuation along with the principal eigenvectors. Graph representing the fluctuations of residues along the first two principal eigenvectors with atom index along the *X*-axis and eigenvector along the *Y*-axis for Ng-WT and Ng-Mut. Ng-WT and Ng-Mut are indicated in black and red, respectively.

Further, we evaluated the cause of such conformational behavior by determining residue-wise level fluctuations along the two principal eigenvectors for the wild-type and mutant systems (Fig. 4). Region-specific displacement of each residue was observed for each of the two principal eigenvectors. The L103E mutation contributed to the fluctuation of the surrounding 96Q, 97R, 98Q, and 99K residues, whereas the L103E residue itself showed little fluctuation. The important residues were observed to interact with DNA in the crystal structure, but some residues, such as K118, Q119, K121, T122, Q124, Q125, R128, and M129, showed large fluctuations in the Ng-Mut system, as shown in Fig. 4. Eigenvector 2 values showed fluctuations of most residues in the Ng-Mut complex (Fig. 4), which may be because of the L103E mutation. This residual fluctuation may account for the divergence in the conformational behavior of both systems.

**Fig. 5:**
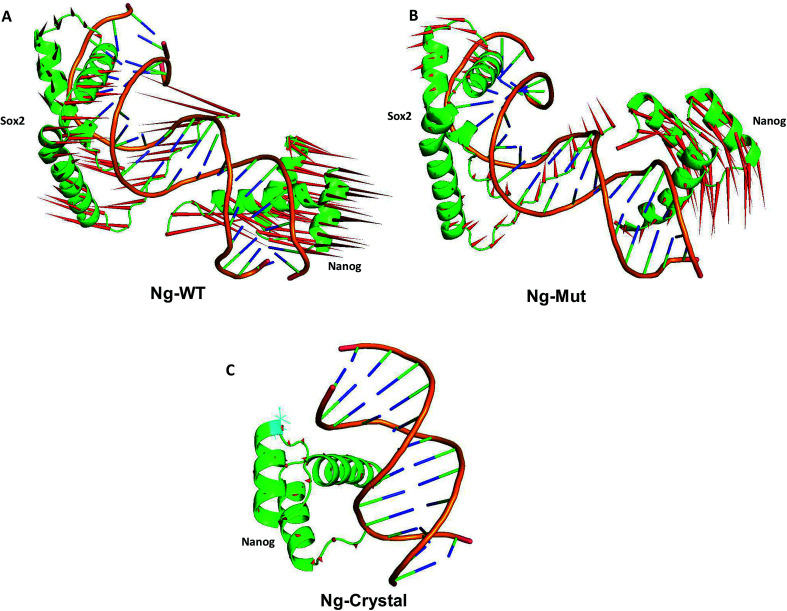
Principal modes of motion for Ng-WT, Ng-Mut and Ng-Crystal. Dominant motions of Nanog and Sox2 in Ng-WT, Ng-Mut, and Ng-Crystal (Nanog only in this case) complexes. The heavy atoms have been used for the analysis; however, projections for the backbone atoms have been displayed for clarity. The magnitudes and directions of motion of the residues are indicated by green arrows in the cartoon structure.

These scattered conformations visited by the Ng-WT and Ng-Mut proteins were further verified by drawing a porcupine graph (Fig. 5). Both the systems displayed similar as well as distinct contradicting movements with respect to each other. The dominant motions displayed by Nanog in Ng-WT and Ng-Mut were found to be similarly oriented, however, the intensity of motion in Ng-Mut was higher towards the DNA. The residues of Nanog showed more harmonious movements in Ng-WT, whereas, such harmony was reduced in Ng-Mut. Sox2 exhibited the similar motion in both the systems, whereas the movement of Sox2 in Ng-Mut complex is less. The residues in the helix 3 region of Nanog showed less movement towards the DNA, enabling non-specific binding. Thus, the L103E mutation may have influenced the essential motions of the surrounding atoms, facilitating their non-specific binding, thereby increasing the stability. In the absence of Sox2, the residual movement of Nanog showed a complete incoherence as indicated by the arrows pointing away from the DNA. This may suggest that the binding of neighboring protein can drastically influence the Nanog binding and functional activity.

### 3.4 Nanog–Sox2 interface on DNA

**Fig. 6:**
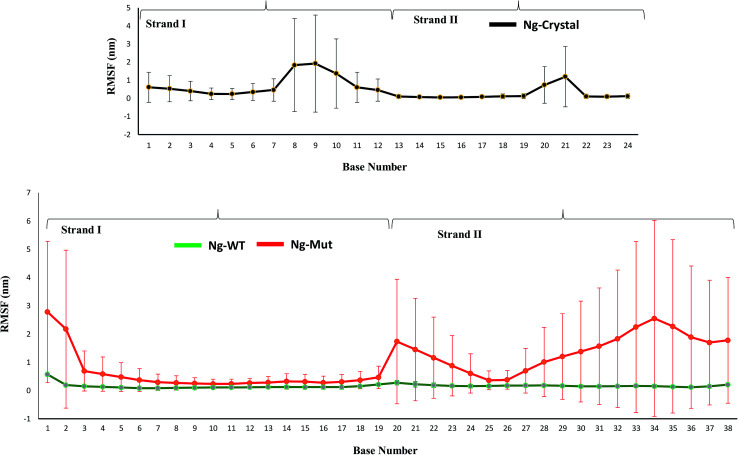
Characteristics of the Nanog–Sox2 interface on DNA. The root mean square fluctuations of the DNA bases for strand 1 (1–19) and strand 2 (20–38) have been displayed. DNA binding site for Ng-WT, Ng-Mut, and Ng-Crystal are shown. Ng-WT and Ng-Mut are indicated in green and red, respectively. The RMSF for Ng-Crystal has been given separately for comparison and is shown in black.

When the proteins (Nanog and Sox2) bound to the DNA, the behavior and orientation of the structure and its dynamics would undergo prominent alterations that can be related to its functionality. The Nanog inserted its 3^rd^ α-helix, H3, into the major groove of its DNA binding site, whereas Sox2 binding was energetically governed by its C-terminal loop. The root mean square fluctuations (RMSF) of the DNA bases (Fig. 6) showed that the strand 1 binding site of Nanog protein in the Ng-WT and Ng-Mut complexes exhibited similar fluctuations as that of the Ng-Crystal. In strand 2, the binding site of Nanog fluctuated more than in the Ng-Crystal. Although strand 2 was not directly linked to Nanog, its movement during simulation was reflected in this RMSF of the Ng-WT and Ng-Mut systems. Hence, the plot provided a view of DNA atom mobility. The presence of protein clearly reduced the mobility of DNA bases within their binding site and the effect was observed as strong for those atoms involved in salt bridges with the protein (Fig. 6).

The protein–DNA interface involving the major third helix was significantly altered in the Ng-WT and Ng-Mut systems compared to the Ng-Crystal. Most interactions disappeared because of Sox2 binding. Except for T122, K125, and M129 residues, the other residues failed to maintain its stronger interactions with its DNA. Hence, it could be understood that the binding of Sox2 altered the stability of Nanog binding to its DNA binding site.

### 3.5 Sox2 influences the dynamics of DNA

The natural tendency for protein binding with DNA alters the DNA conformational states. The conformational changes in DNA were evaluated by various DNA parameters calculated using CURVES+.^28^

Sox2 is known to bend DNA, and examination of protein-induced DNA bending is necessary to gain insight into DNA's structural deformation. As a result of this bending nature, the binding of Sox2 altered the conformation of B-form DNA into the non-standard B-form (or A-form). DNA can be classified based on various parameters such as twist (A = 33; B = 36), rise (A = 2.56A; B = 3.38A), roll (A = 6; B = 0) and slide (A < −0.8; B > −0.8)^32,33^ (Fig. S8†). The crystal structure without Sox2 showed an average bending angle of approximately 22°, whereas Ng-WT and Ng-Mut possessing Sox2 adjacent to the Nanog binding site showed a bend angle of approximately 50–60°. The bend angle was lower in Ng-WT initially, however, towards the end of simulation, both complexes showed a bend angle of similar range. The binding of Sox2 adjacent to Nanog caused the DNA to be in a nonlinear state. Even though the average bending value in Ng-Mut (∼55) was higher than Ng-WT (∼50), the fluctuation was quite less than the other.

### 3.6 Configurational entropy

The entropy of a bio-molecule plays an important role in determining the physical and chemical phenomena of a system. A major limitation in a computer simulation is the estimation of absolute entropies and entropy differences.^34^ However, various approximation approaches, such as quasi-harmonic analysis, show good agreement with experimental observations.^27^ Quasi-harmonic analysis has been demonstrated to provide a combination of qualitative and quantitative information about the dynamic behavior of protein/DNA in MD simulations. This method is useful for estimating changes in configurational entropy in a complex system (protein and DNA), and may, therefore, contribute to our understanding of the thermodynamics of biomolecular interactions. Since the system moves in all dimensions and the movement range is not restricted by periodic boundary conditions, the quasi-harmonic approximation would provide correct changes in entropy.^34^

The entropy of the protein–DNA complex was calculated by superposition of all frames using the heavy atoms (non-hydrogen) atoms as a reference. The entropy has been extracted from the covariance matrices using quasi-harmonic approximation after removing rotational and translational movement. The entropy values of all three systems were increasing until it reaches a plateau (Fig. S9†). The configurational entropy values were higher in Ng-WT and Ng-Mut compared to the crystal structure that may be due to the number of atoms involved (Ng-Crystal system lacks Sox2). The specific tight binding of the Nanog in the crystal structure required less configurational subspace, whereas Sox2 binding influenced the Nanog-bound structure by recruiting a larger configurational subspace, resulting in significantly higher entropy values.

## 4 Discussion

Sox2 influences the orientation and dynamics of the DNA-bound configuration of other TFs including Nanog.^35^ This mutual interaction can affect the induction of downstream genes. Therefore, it is worthwhile to study these interaction in detail and to pursue this computational analysis is leveraging a great support. The simulation of these complexes has been repeated three times, and the final/average results have been discussed here. The Nanog and Sox2 interaction have been delineated in this study, and based on our analysis, we found that the influence of Sox2 on Ng-Mut was greater than that on Ng-WT, which is correlating the fact that Ng-Mut complex is more efficient than the Ng-WT. In protein–DNA interaction, the charged residues play important roles, and the charge distribution over protein has been widely studied that alter protein–DNA binding. In Nanog, the mutation of L103E replaces a non-polar residue by the negatively-charged residue introducing electrostatic interactions between Nanog and DNA, thereby experiencing higher number of interaction than WT (Table 1). Moreover, from structural viewpoint, leucine and glutamate have comparable helix propensity values (*L* = 1.21 *vs. E* = 1.51) resulting in less structural influence over Nanog (Fig. 2).

Although the binding of Sox2 in both cases (Ng-WT and Ng-Mut) influenced the binding orientation of Nanog with its DNA and sampled less binding energy, the complex remained aligned with its native structure *via* protein–protein and protein–DNA interactions (Fig. 2, 3 and Table 1). The Ng-Mut complex is maintaining a single hydrogen bond between Nanog and Sox2 (Arg76 of Sox2 with Gln135 of Nanog), whereas the Ng-WT failed to do so (Table 1). In addition to that, Hayashi *et al.*, studied the Nanog L122A mutation which enhance the DNA binding affinity in Oct4 promoter region brings up the fact that the mutation of Lys122 is very much important for Nanog reprogramming.^11^ Similarly, our mutant complex (L102E) was showing the better affinity with its binding partner as well as DNA (Fig. 5 and Table 1); however, both the complexes are existing with good number of interaction with their DNA (Table 1).

Differential responses of Ng-WT and Ng-Mut because of Sox2 binding may be correlated to the localized protein motions when the systems were analyzed using PCA. The internal motion of Ng-WT was limited to a subspace with fewer dimension compared to Ng-Mut, whereas the internal motion of the crystal structure was negligible (Fig. 3C). The Ng-WT and Ng-Mut systems largely remained in one conformational space indicating lower energy, while the infrequent transition to different space for other conformations, though fewer, but have been observed for these complexes (Fig. 3A and B). The porcupine graph shows that the protein dominant movements in the Ng-WT and Ng-Mut systems were different; the protein residue movements in Ng-Mut were more coherent with lower magnitude, and the movements of Nanog residues were supportive to each other in DNA binding, whereas, Ng-WT showed higher degree of residual movement away from DNA (Fig. 5). The L103E mutation may have influenced the essential motions of the surrounding atoms, facilitating non-specific binding of Nanog and increasing stability (Fig. 4). Similarly, non-specific binding of lactose repressor showed fluctuating residual movements,^36,37^ suggesting that Sox2 alters the specific binding to become non-specific.

The RMSF of the DNA bases dynamics decreased in the presence of Sox2 protein molecule (Fig. 6). However, the configurational entropy of the Ng-WT and Ng-Mut systems revealed higher energy requirements compared to the Ng-Crystal system (Fig. S9†), suggesting that the Sox2–Nanog bound DNA structure and its transcriptional regulation is achieved through concerted modulation of DNA-mediated interactions.

The largest changes in the conformational entropy of a protein arise from the energetic restraints from the backbone and side chain groups.^34^ The configuration of Ng-Crystal system is lower due to the absence of Sox2, thus, the entropy of the simulated crystal structure was lower than those of the model systems (Ng-WT and Ng-Mut) (Fig. S9†). Although, it is almost impossible to calculate the absolute entropic values, however, a qualitative observation can be useful to assign the energetic state to each molecule. Ng-Mut has slightly more entropy that allowed the complex to visit more states, and this can be attributed to the addition of a charged residue. As the entropy difference is no more than 1 kcal mol^−1^ K^−1^, both systems essentially visited similar meta-stable states.

Cooperative binding of Sox2 forced the system to use more configurational subspace and energy. The helix–turn–helix region of the HMG domain bent the DNA to approximately 50–60° and the bending nature of the Sox2 molecule is necessary for its activity,^38^ and to provide better stability.^14,39^ Ng-Crystal showed a constant bending angle, whereas Nanog bound to Sox2 showed an increased bending angle, revealing that the systems require a stable bending angle to stabilize the conformation compared to the DNA-bound structures; additionally, apart from this DNA-mediated protein–protein interaction, the system may require a DNA-independent interaction.^6^

The Ser-rich region and the transactivation domain of Nanog are unconstrained regions, where modeling and simulating the whole length TAD protein is nearly impossible and may require very long simulation time with supercomputing facilities. We tried to model this domain, however, lack of suitable template and abundance of non-structured region hindered its proper modeling. Therefore, a low confidence protein model might have a spurious effect in simulation and may invalidate the overall conclusion of this study. Moreover, a common way for the DNA binding proteins to interact is through non-DNA binding domains and this has been reported for Nanog–Sox2 interaction as well.^6^ Both, the Sox2 and Nanog have multiple domains such as HMG, transactivation domain, and Ser-rich motifs for Sox2, and DNA binding domain, transactivation domain, and Trp repeats for Nanog. The experimental evidence suggest that the major interaction between Nanog and Sox2 is governed by the sequences of non-DNA-binding domains (through the transactivation domains). However, the SELEX (Systematic evolution of ligands by exponential enrichment) results suggested that the interaction occurs in a specific spatial relationship of the DNA-binding domains of these proteins.^10^ Sox2 can interact with Oct4 over the DNA as reported by Merino *et al.*, 2014,^40^ so the role of DNA-based interaction cannot be ruled out in case of Sox2–Nanog interactions.^41^ Moreover, the bending of DNA by Sox2 has drastic influence over the ability of transcription factors to induce transcription of target genes.^38^ Other findings that the bending or conversion to non-standard DNA is desirable and supports the notion that Sox2 not only interacts through its TAD but also facilitate the Nanog transcriptional ability by indirect physical interaction through bending of DNA. As it has already been reported that the DNA-independent interaction is governed by the transactivation domain of Sox2 and tryptophan repeat (WR) domain of Nanog,^6^ these two proteins may facilitate better and stronger binding *via* fewer DNA-mediated interactions, which is driven by their DNA-binding domains of the proteins. The overall interactions and the energy profile obtained for Ng-Mut is favoring for its better stability than the Ng-WT, which is correlating with the experimental data. Our MD simulation results explains the interactions between Nanog and Sox2 through their DNA-binding domains, and suggest that, despite of this weaker DNA-independent interaction profile, Nanog–Sox2 cooperativity *via* DNA-binding domains are also necessary for its better and stable interaction profile.

## Author contributions

DY designed and performed experiments. DY and MAA analyzed the results. DY and SC wrote the manuscript.

## Conflicts of interest

There are no conflicts of interest to declare.

## Supplementary Material

RA-009-C8RA10085C-s001
