# Supplementary material for: Structural mechanism of DNA-mediated Nanog–Sox2 cooperative interaction
Source: RSC Adv. 2019 Mar 13;9(14):8121–30. doi: 10.1039/c8ra10085c (PMC9061787; doi:10.1039/c8ra10085c)
Supplement: RA-009-C8RA10085C-s001 [file RA-009-C8RA10085C-s001.pdf]

Electronic Supplementary Material (ESI) for RSC Advances.
This journal is © The Royal Society of Chemistry 2019

<Supplementary Information>

Structural mechanism of DNA-mediated Nanog-Sox2 cooperative interaction

Dhanusha Yesudhas, Muhammad Ayaz Anwar and Sangdun Choi*

Department of Molecular Science and Technology, Ajou University, Suwon, 16499, Korea

***Correspondence**

Sangdun Choi

Department of Molecular Science and Technology, Ajou University, Suwon, 443-749, Korea

Fax: +82 31-219-1615

Tel: +82 31-219-2600

E-mail: sangdunchoi@ajou.ac.kr

Running Title: *In silico* analysis of Nanog-Sox2 mediated cooperativity in transcription

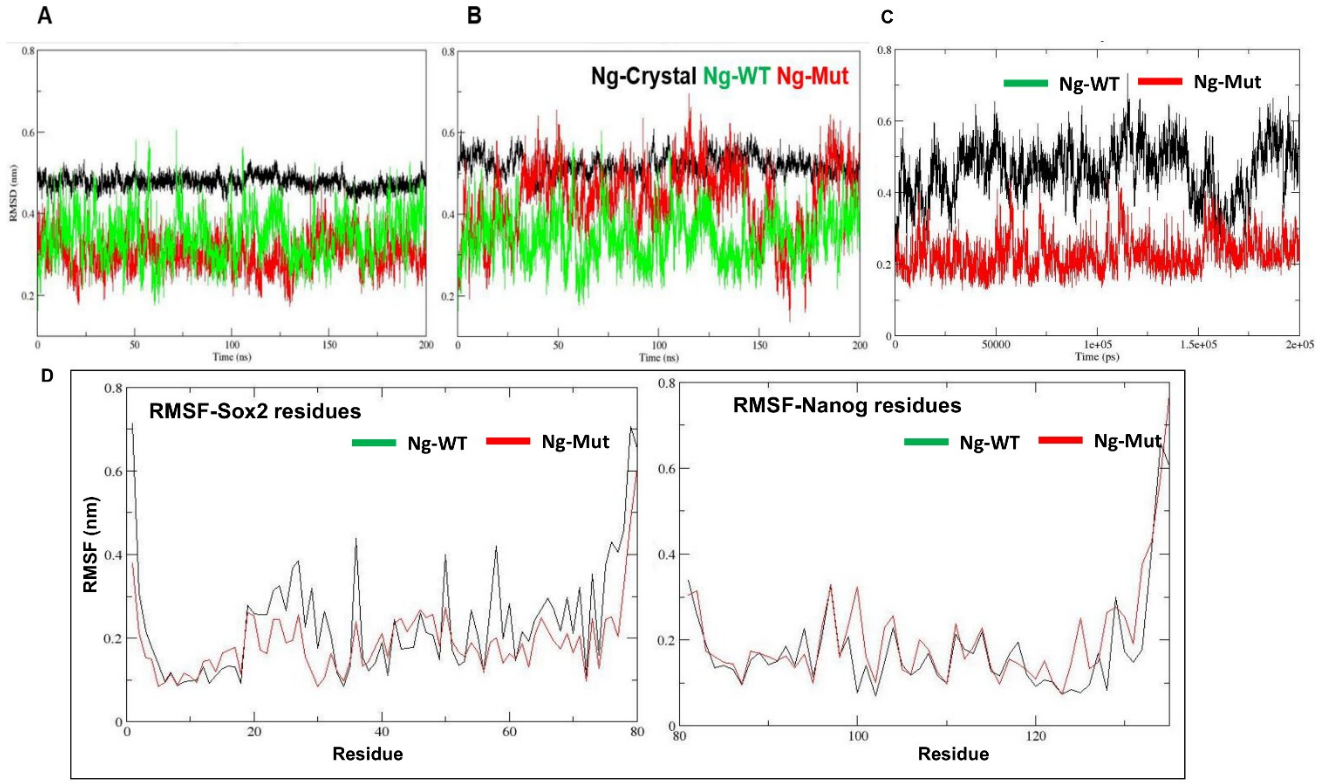

Fig. S1 | Root mean square deviation (RMSD) and Root mean square fluctuation (RMSF) for Ng-WT, Ng-Mut, and Ng-Crystal. RMSD of the backbone carbon atoms for Ng-WT, Ng-Mut, and Ng-Crystal are showing the overall fluctuations throughout the simulation. A, B and C refers to three different independent simulations for these systems. (D) The representation of RMSF fluctuations of Nanog and Sox2 residues in Ng-WT and Ng-Mut complexes.

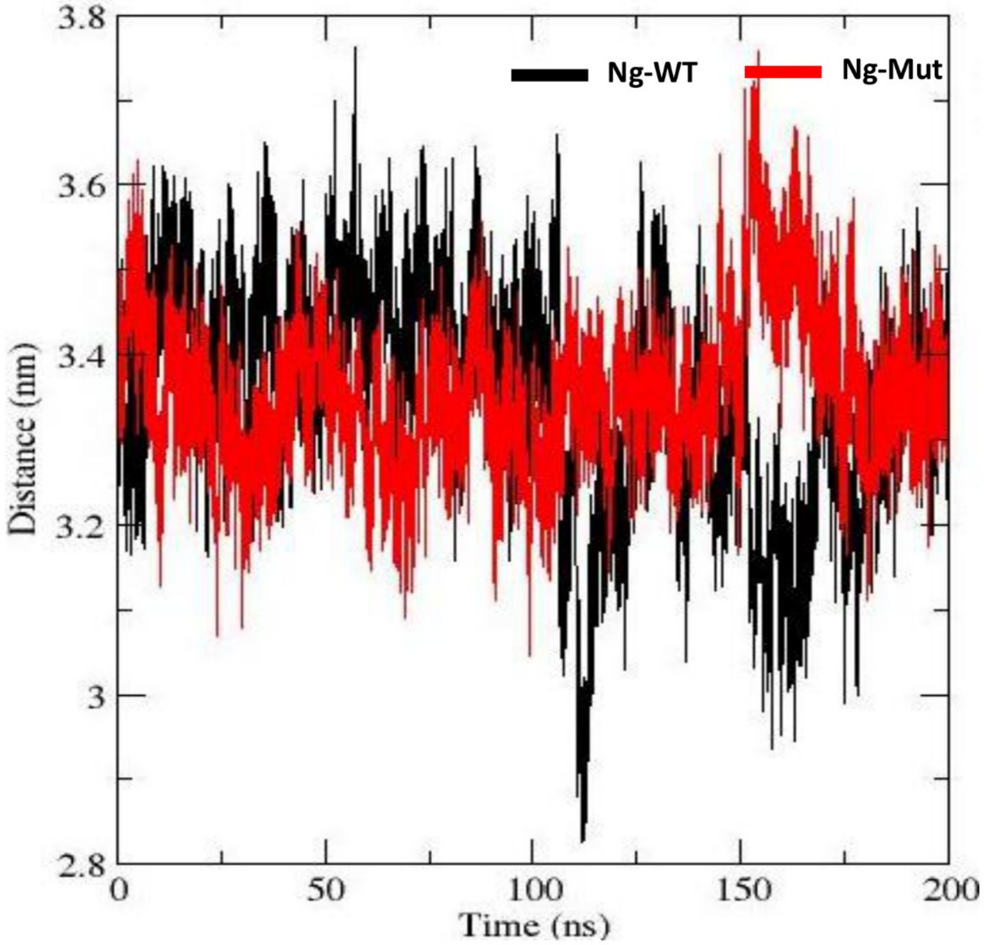

Fig. S2 | Minimum distance between Nanog and Sox2 in Ng-WT and Ng-Mut. (A) Representation of minimum distance between Nanog and Sox2 in Ng-WT and Ng-Mut with simulation time along the X-axis and distance along the Y-axis.

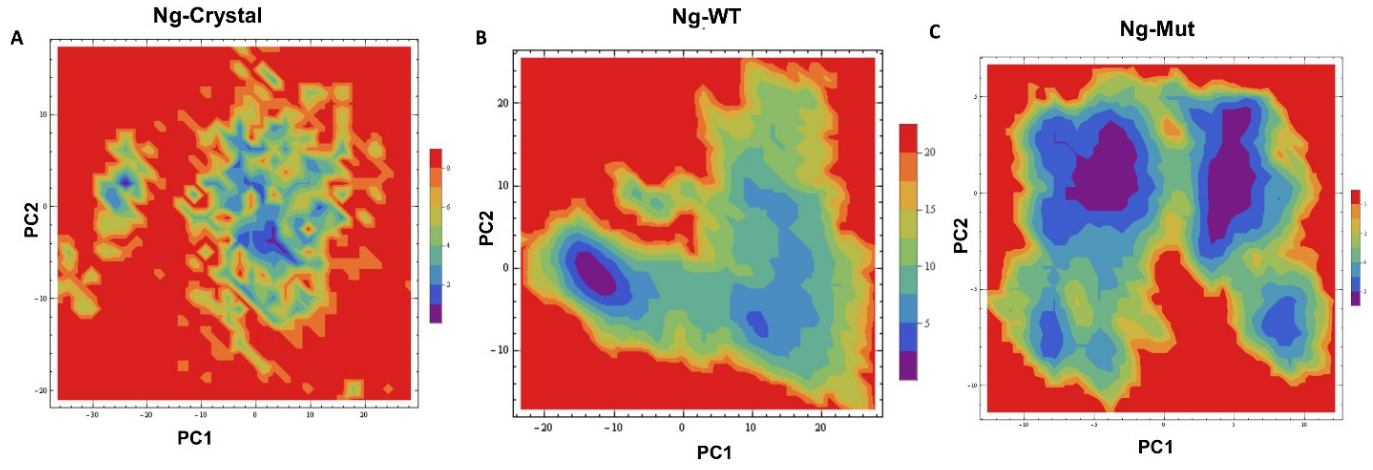

Fig. S3 | Free energy landscape (FEL). FEL representation along with the energy values are displayed for Ng-crystal (A), Ng-WT (B) and Ng-Mut (C) complexes.

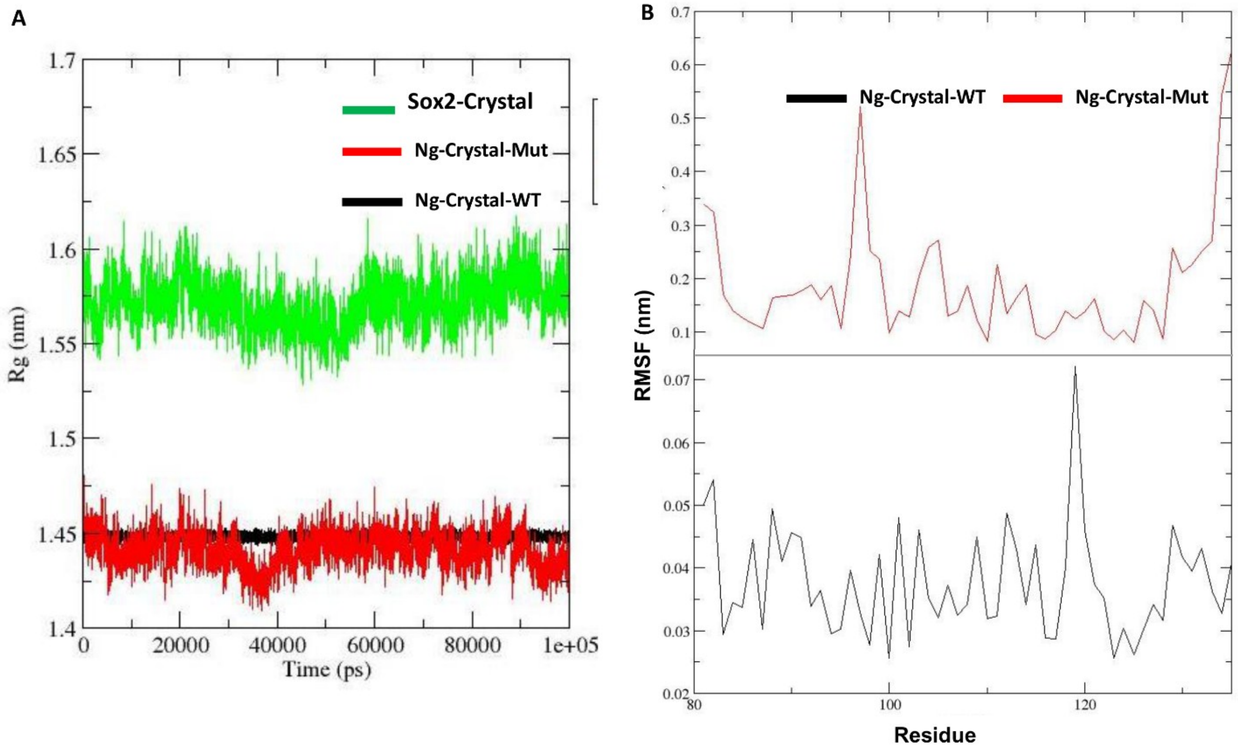

Fig. S4 | Radius of gyration and RMSF of Ng-Crystal-WT/Mut (A) Representation of radius of gyration in all three crystal structure, displays the Ng-Crystal-WT has better and compact conformation. (B) Representation of Nanog residues fluctuations in Ng-Crystal complexes.

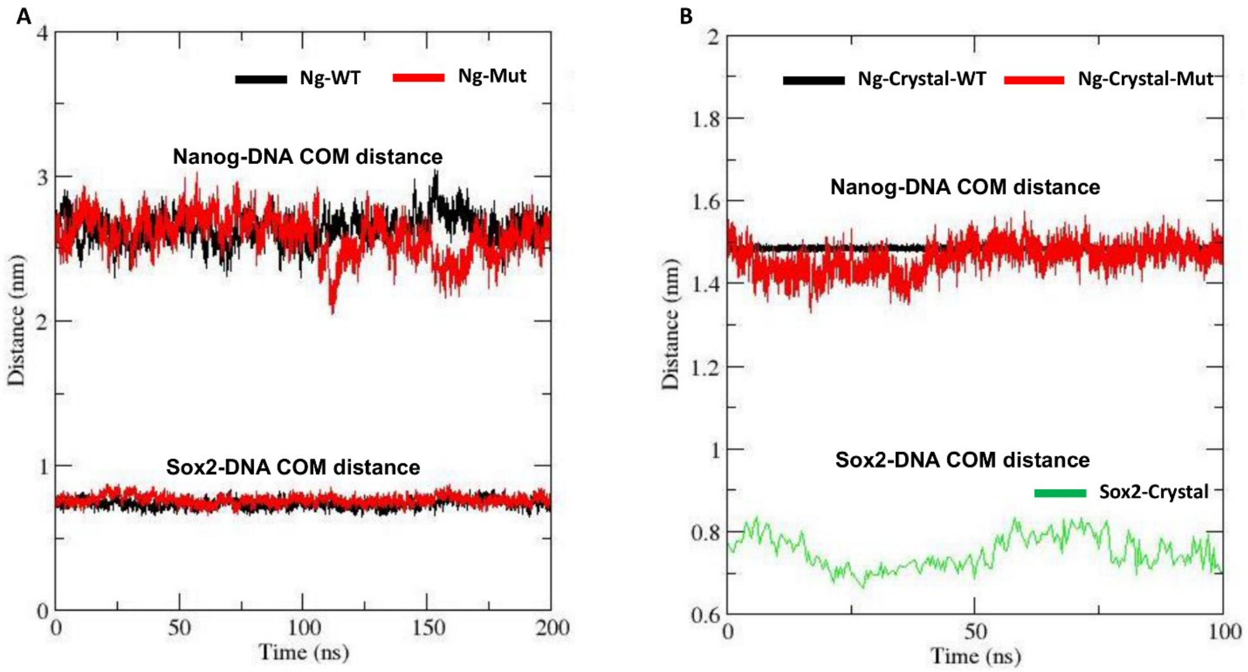

Fig. S5 | Distance between the proteins and DNA (A) Representation of distance between Nanog and DNA as well as Sox2 and DNA in Ng-WT and Ng-Mut with simulation time along the X-axis and distance along the Y-axis. (B) Representation of distance between Nanog and DNA in Ng-Crystal-WT and Ng-Crystal-Mut complexes, along with the Sox2-DNA distance in the Sox2-crystal complex.

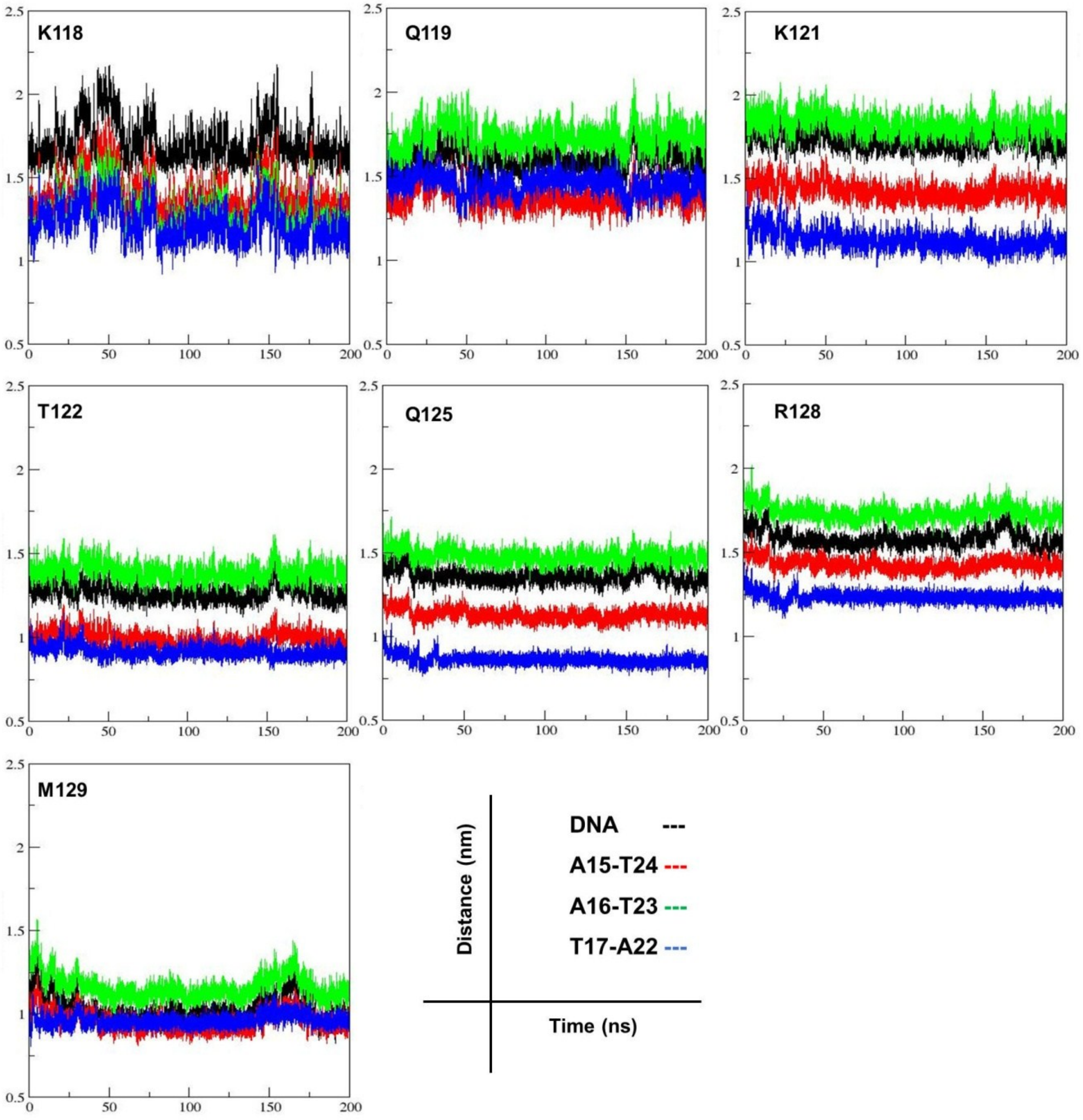

Fig. S6 | Residual movement of Ng-WT with respect to DNA sequence. Representation of the position of helix 3 residues K118, Q119, K121, T122, Q125, R128 and M129 with respect to the plane of DNA bases with simulation time along the X-axis and distance along the Y-axis.

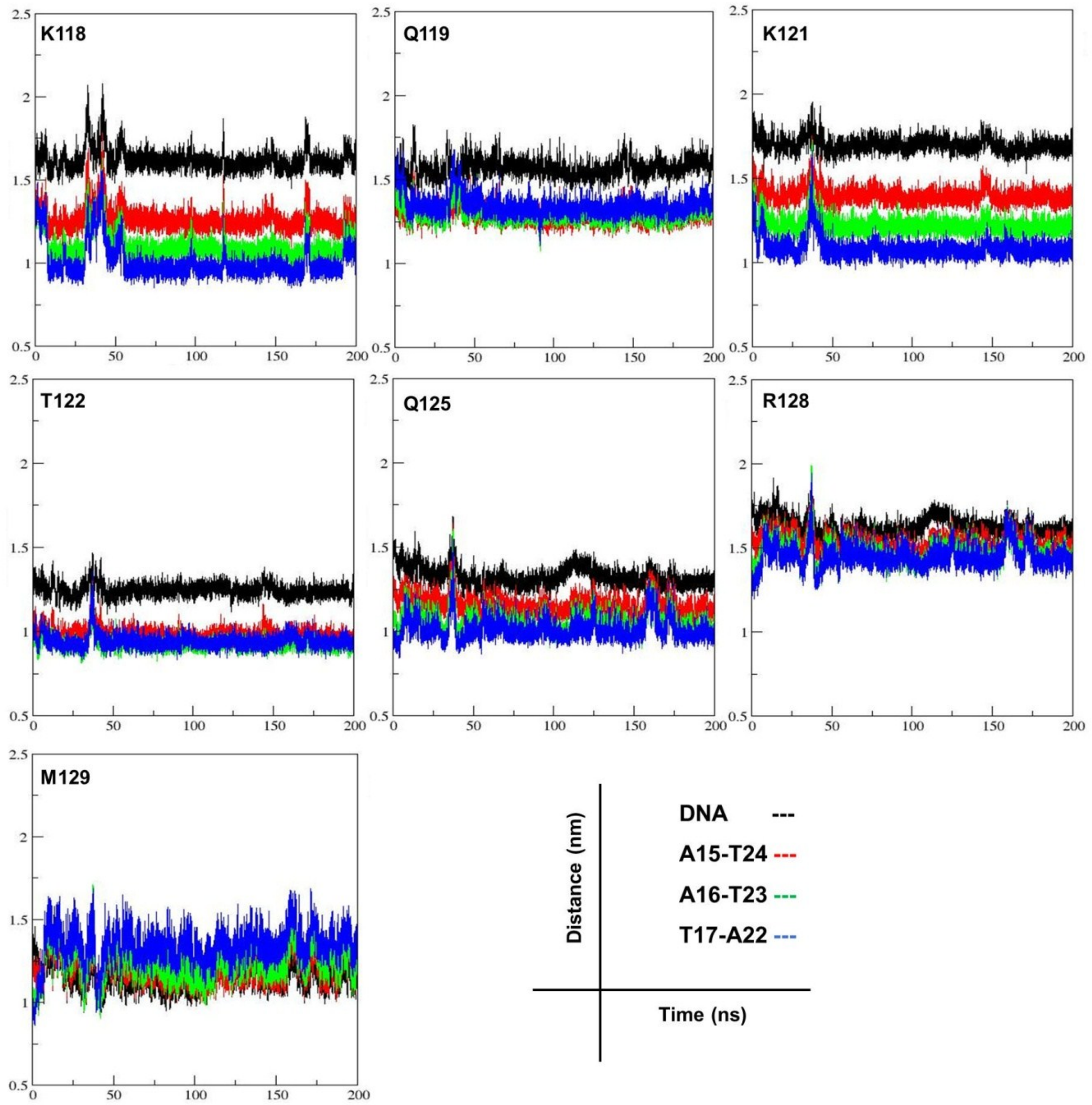

Fig. S7 | Residual movement of Ng-Mut with respect to DNA sequence. Representation of the position of helix 3 residues K118, Q119, K121, T122, Q125, R128, and M129 with respect to the plane of DNA bases with simulation time along the X-axis and distance along the Y-axis.

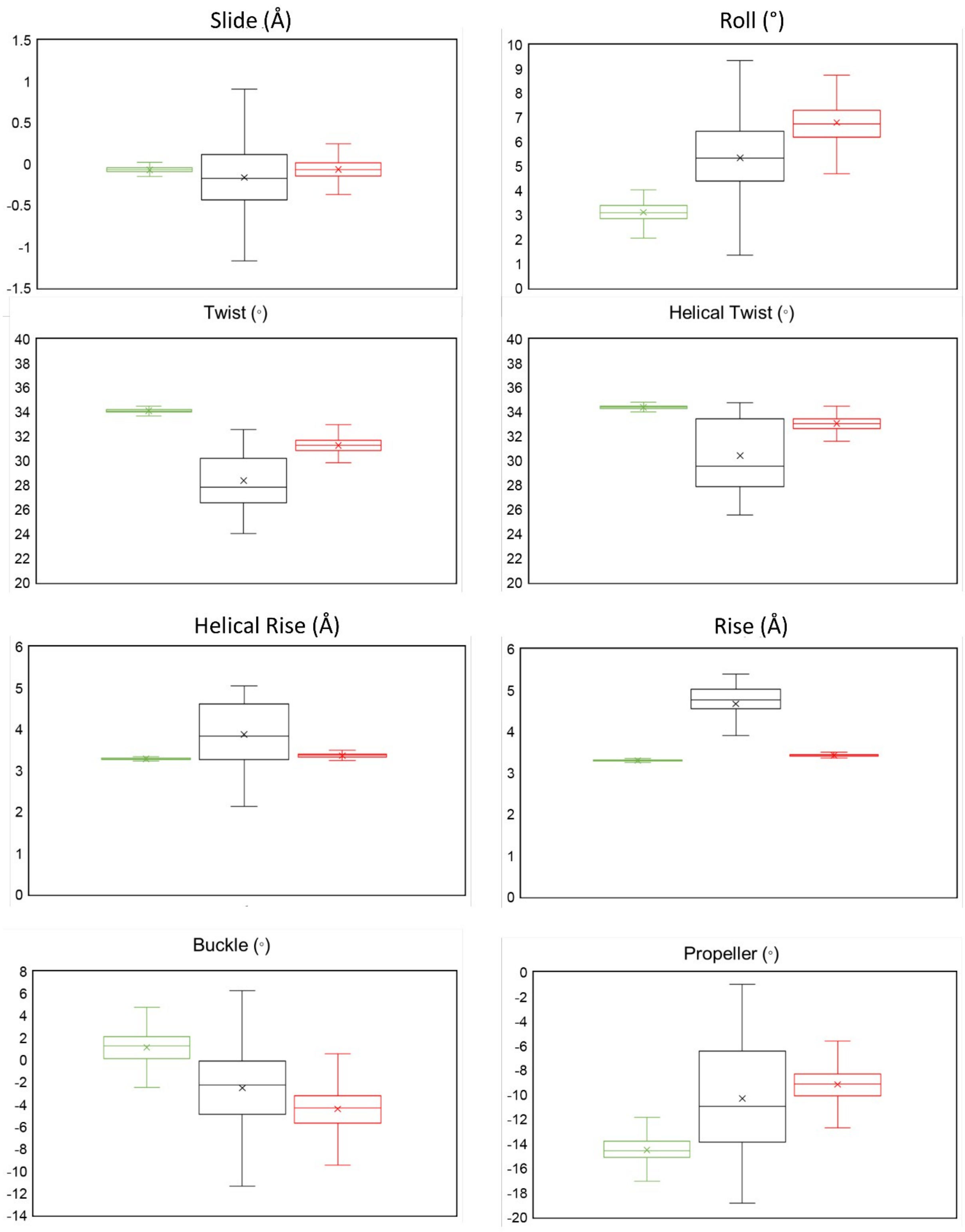

Fig. S8 | Comparison of DNA parameters. Various DNA parameters describing the A-DNA or

B-DNA have been presented. The data of DNA parameters from Ng-Crystal, Ng-WT and Ng-Mut are given in green, black and red respectively.

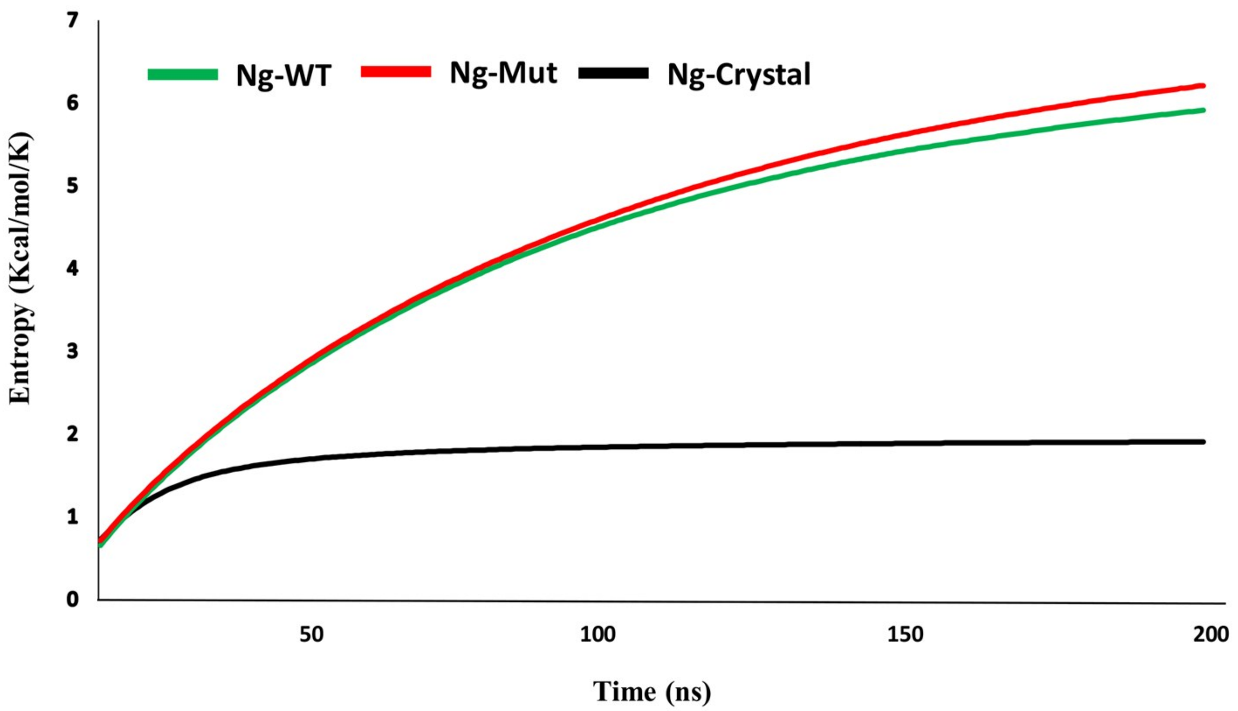

Fig. S9 | Quasi entropy values. The entropy energy values for Ng-WT, Ng-Mut, and Ng-Crystal calculated using quasi harmonic approximation method are plotted as a graph with the simulation time along the X-axis and entropy energy values along the Y-axis. The graph is potted based on the average values from three independent simulations.

Ng-crystal-WT					Ng-Crystal-Mut				
TYR37	HE2	DG2	O1P	3.273	TYR117	OH	DG2	O1P	2.626
LYS41	HD2	DG2	H2'	2.989	LYS121	HE1	DG2	O5'	3.147
ARG48	HH2	DC3	P	3.422	ARG128	HH2	DC3	O2P	2.982
TYR20	HE2	DC3	H3'	3.194	GLN125	CD	DC3	H3'	3.481
GLN45	OE1	DC3	H3'	2.809	TYR100	HE2	DC3	O3'	2.939
GLN45	HE2	DC4	H2'	3.363	MET129	HE3	DC4	H3'	3.392
LYS52	NZ	DC5	P	3.121	LYS132	NZ	DC4	H3'	3.392
THR1	O	DA17	H4'	2.214	TRP123	HE1	DA17	O2P	3.112
ASN46	HD2	DA17	C8	3.105	PHE83	CE2	DA17	O5'	3.373
GLN39	HE2	DA18	O2P	3.454	THR81	O	DA17	H4'	2.813
THR42	CG2	DA18	H8	3.487	ASN126	HD2	DA17	C8	3.281
LYS38	HZ2	DT19	O2P	3.397	GLN119	HE2	DA18	P	3.308
**LYS21**	**OE1**	**DC**	**H3'**	**3.212**	THR122	HG2	DA18	H8	2.901
					ASN126	HD2	DA18	H8	3.163
					**GLU103**	**OE1**	**DC3**	**H3'**	**3.112**
					THR122	HG2	DT19	H71	3.301

Table S1 | Protein-Protein interacting residues in Ng-Crystal complexes. The interacting residues of Nanog with the base pairs interactions have been tabulated, and the interacting distance is in Å. The mutant residue interaction has been marked in bold.
